# Stress-responsive gene *TauT* and acute kidney injury

**DOI:** 10.1186/1423-0127-17-S1-S28

**Published:** 2010-08-24

**Authors:** Xiaobin Han, Russell W Chesney

**Affiliations:** 1 Department of Pediatrics, University of Tennessee Health Science Center, and the Children's Foundation Research Center at Le Bonheur Children's Medical Center, Memphis, Tennessee, USA

## Abstract

**Background:**

Cisplatin is a commonly used chemotherapeutic agent that has a major limitation because of its nephrotoxicity. We have demonstrated that cisplatin down-regulates the expression of the taurine transporter gene (*TauT*) in renal cells and that forced overexpression of *TauT* protects against cisplatin-induced apoptosis in renal cells *in vitro* and *in vivo*. In the present study, we have investigated how *TauT* is regulated by p53 and c-Jun and its role during acute kidney injury (AKI).

**Methods:**

Regulation of *TauT* by p53 and c-Jun was determined by reporter gene assay, DNA binding, Western blot analysis, and immunohistochemistry.

**Results:**

*TauT* was down-regulated by p53 and up-regulated by c-Jun. Two potential binding sites for c-Jun were identified in the promoter region of *TauT*. Inhibition of c-Jun N-terminal kinase (JNK) enhanced *TauT* promoter activity. Overexpression of *TauT* protects against cisplatin-induced kidney injury in a *TauT* transgenic mouse model.

**Conclusions:**

Our findings suggest that *TauT* plays a critical role in renal function. Expression of *TauT* is negatively regulated by p53 and positively regulated by c-Jun, which is mediated by the JNK signaling pathway. The outcome level of *TauT* may determine the fate of renal cells during stress-induced AKI.

## Background

Acute kidney injury due to ischemic or toxic renal injury is a common disorder with a mortality of about 50% [1,2]. A vast majority of research in the field has focused on the determination of events and factors that cause renal proximal tubular cell (RPTC) injury and death and lead to the development of AKI. Cisplatin-induced AKI is currently a topic of intense study. As a highly effective chemotherapeutic agent, cisplatin has been used to treat a wide variety of solid tumors [3]. However, 25-35% of patients experience a significant decline in renal function after the administration of a single dose of cisplatin [4]. Several mechanisms, including oxidation, inflammation, genotoxic damage and cell cycle arrest, have been implicated in cisplatin nephrotoxicity [5-10]. The JNK (c-Jun N-terminal kinase) pathway is a major stress signaling pathway in cells that plays important roles in many cellular processes, including development, apoptosis, cell growth and immune responses [11,12].

Recently, the mechanisms of renal cell repair and regeneration have garnered much attention [7]. Unfortunately, the development of therapeutic strategies that are efficacious in humans with AKI has proven problematic. This suggests that the development of more successful therapies requires approaching the problem from a different vantage point [1]. The regenerative capacity of the kidney is well documented [2], and the responses of surviving RPTC are thought to be crucial to the restoration of renal function following AKI. Consequently, identifying genes that are involved in RPTC protection, repair, and regeneration may uncover new therapeutic targets that promote renal recovery and decrease the severity of AKI.

## Methods

### Cell culture

MDCK kidney cells were cultured according to AATC (American Association Tissue Culture) guidelines. Briefly, cells were grown as confluent monolayers in 10 cm diameter tissue culture plates in DMEM media specific for each cell line with 10% fetal calf serum at 37°C in the presence of 5% CO_2_ in a humidified incubator. Cells were plated 18 h before transfection and fed with fresh medium 4 h before transfection.

### Construction of the reporter gene

The promoter region of *TauT* was identified in previous studies [13], and a p53-binding consensus site was found in the *TauT* promoter sequence, located at -663 to -695. In this study, ~1.1 kb of the TauT promoter region DNA was used as the template for PCR (GenBank^TM^/EBI accession number AR151716) and the PCR fragment was cloned into the promoter-less luciferase vector pGL3-Basic (Promega, Madison, WI) to generate the plasmid p963 for use in transfections and luciferase assays. The conditions used were 30 cycles of 1 min of denaturation at 94°C, 1 min of annealing at 58°C, and 1 min of elongation at 72°C. The sense primer (5'-GGGGTACCTTACTGAAGGTCACACAGC-3') designed for PCR contained a unique site for KpnI, and the antisense primer (5'-AAGATCTTGGCACGGGAGTTCA-3') contained a unique site for BglII. PCR products were digested with KpnI and BglII and re-ligated into KpnI and BglII sites of pGL3-Basic to generate plasmids containing segments of the *TauT* promoter sequence extending from the +48 nucleotide corresponding to the transcriptional start site. The constructs were verified by DNA sequencing. The p53-binding site deletion (del pGL-563) and p53 mutation (mt pGL-963) constructs were generated from the p963 plasmid by using sense primers 5'-GGGGTACCGAGTTGGGGAGGGA-3', and 5'-GGGGTACCAGATGAGG-AAACCCCCACACAGAAGGTCTGGGGCTTGCCTGATGTCA-3', respectively. The antisense primer used for these constructs was the same as described above.

### Transient transfection

Plasmid DNA was introduced into cultured MDCK cells using cationic liposomes (LipofectAMINE). Transfection was carried out for 16-18 h, and then cells were washed twice with phosphate-buffered saline and incubated in fresh medium for 24-48 h before harvesting. pGL-control, which contains a luciferase gene driven by the SV40 early region promoter/enhancer, and empty pGL-Basic vectors were used as positive and negative controls, respectively. To standardize the transfection efficiency, 0.1 µg of pRL-CMV vector (pRL *Renilla reniformis* luciferase control reporter vector; Promega) was cotransfected in all experiments. Cells were harvested 48 h after transfection and lysed in 200 µl of reporter lysis buffer (Promega). A luciferase assay was performed using a dual luciferase assay kit (Promega), and activity was measured with an Optocomp 1 luminometer (MGM Instruments, Inc., Hamden, CT). Promoter activity (mean ± S.D. of four samples in relative light units) of each construct is represented by relative light output normalized to pRL-CMV control. Graphs represent typical results of four separate experiments. The concentration of protein in the cell extracts was determined using the Bradford method (Bio-Rad, Hercules, CA).

### Measurement of taurine transport

Taurine transport studies were performed on confluent monolayers three days after seeding cells. Briefly, cells were washed with Earle’s Balanced Salt Solution (EBSS) at 37°C. Uptake was initiated by the addition of uptake buffer (2 mM KCl, 1 mM MgCl_2_, 96 mM NaCl, 1.8 mM CaCl_2_, 5 mM Hepes, pH 7.6) to which 50 µM unlabeled taurine and 0.5 µCi/ml ^14^C-taurine (Perkin Elmer, Boston, MA) were added. After incubation for 30 min at room temperature, uptake was terminated by the removal of uptake buffer followed by three rapid washes with cold EBSS. Cells were solubilized in 1% SDS in 0.2 N NaOH and radioactivity was counted in a Packard 2000-CA Liquid Scintillation Analyzer.

### *In vivo* model of cisplatin-induced AKI

Male mice (wild-type and *TauT* transgenic), 10-12 weeks old and weighing 28 to 30 g, were assigned to treatment groups (n = 8/group). For the experiment, eight *TauT* transgenic mice and eight wild-type mice received a single dose of cisplatin (15 mg/kg body weight) by intraperitoneal injection. Eight saline-injected mice were used as controls. To determine cisplatin-induced nephrotoxicity, mice were sacrificed three days after cisplatin injection. Kidney samples were collected and treated in 10% buffered formalin until used.

### Western blot analysis

Cells were lysed in 50 µl M-PER mammalian protein extraction reagent (Pierce, Inc., Rockford, IL) supplemented with a protease inhibitor cocktail for use with mammalian cell and tissue extracts (Sigma). The lysates were cleared by centrifugation at 14,000 x *g* for 2 min, and the supernatants were transferred to clean tubes. Equal amounts of protein (50 µg) were separated by electrophoresis on a 12% SDS-polyacrylamide gel and transferred to a nitrocellulose membrane (Millipore, Bedford, MA) using a semi-dry electrophoretic transfer system (Bio-Rad). Membranes were incubated in 5% nonfat dry milk in Tris base/sodium chloride (TBS) buffer with 0.2% Tween 20 (TBST) at 4°C overnight. The membranes were incubated with primary antibodies for 1 h at room temperature. Blots were washed with TBST and incubated with horseradish peroxidase-linked secondary antibody (Sigma) for another hour, and then proteins of interest were detected using a chemiluminescence detection kit (Pierce, Inc.).

### Immunohistochemistry

Immunohistochemistry was performed by following the manufacturer’s instructions (Pierce). Briefly, samples were rehydrated in decreasing ethanol series (100%, 95%, and 70%) for 5 min each. Samples were immersed in 1x PBS for 5 min at room temperature, then quenching solution (3% H_2_O_2_ in methanol) for 5 min, and then washed twice in dH_2_O for 10 min. Slides were blocked for 20 min with the blocking buffer. Primary antibodies (antibody against taurine transporter protein) were applied to slides and incubated for 1 h. Slides were washed for 10 min with PBS, then the biotinylated secondary antibody was applied and incubated for 1 h. After washing for 10 min with PBS, ABC reagent was applied for 30 min. Finally, immunostaining was detected by using a Metal Enhanced DAB Substrate Kit (Pierce).

### Assessment of apoptosis

Apoptotic cells in kidney sections were detected by the terminal deoxynucleotidyl transferase (TdT)-mediated digoxigenin-deoxyuridine nick-end labeling (TUNEL) method following the manufacturer’s instructions (R&D Systems, Minneapolis, MN).

### Statistics

All experiments were performed in triplicate. Luciferase assays are expressed in units of relative light output. The data represent the mean ± standard error of three or four experiments. Statistical comparisons were made using one-way analysis of variance and Student's *t* test to determine significant differences in the means.

## Results

### *TauT* is directly regulated by c-Jun in renal cells

**Figure 1:**
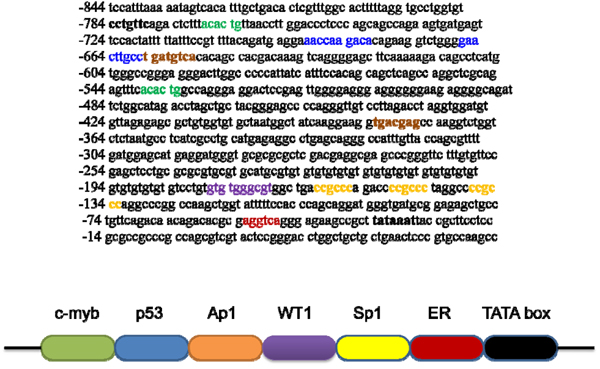
*TauT* promoter sequence and potential binding sites for indicated transcription factors.

**Figure 2:**
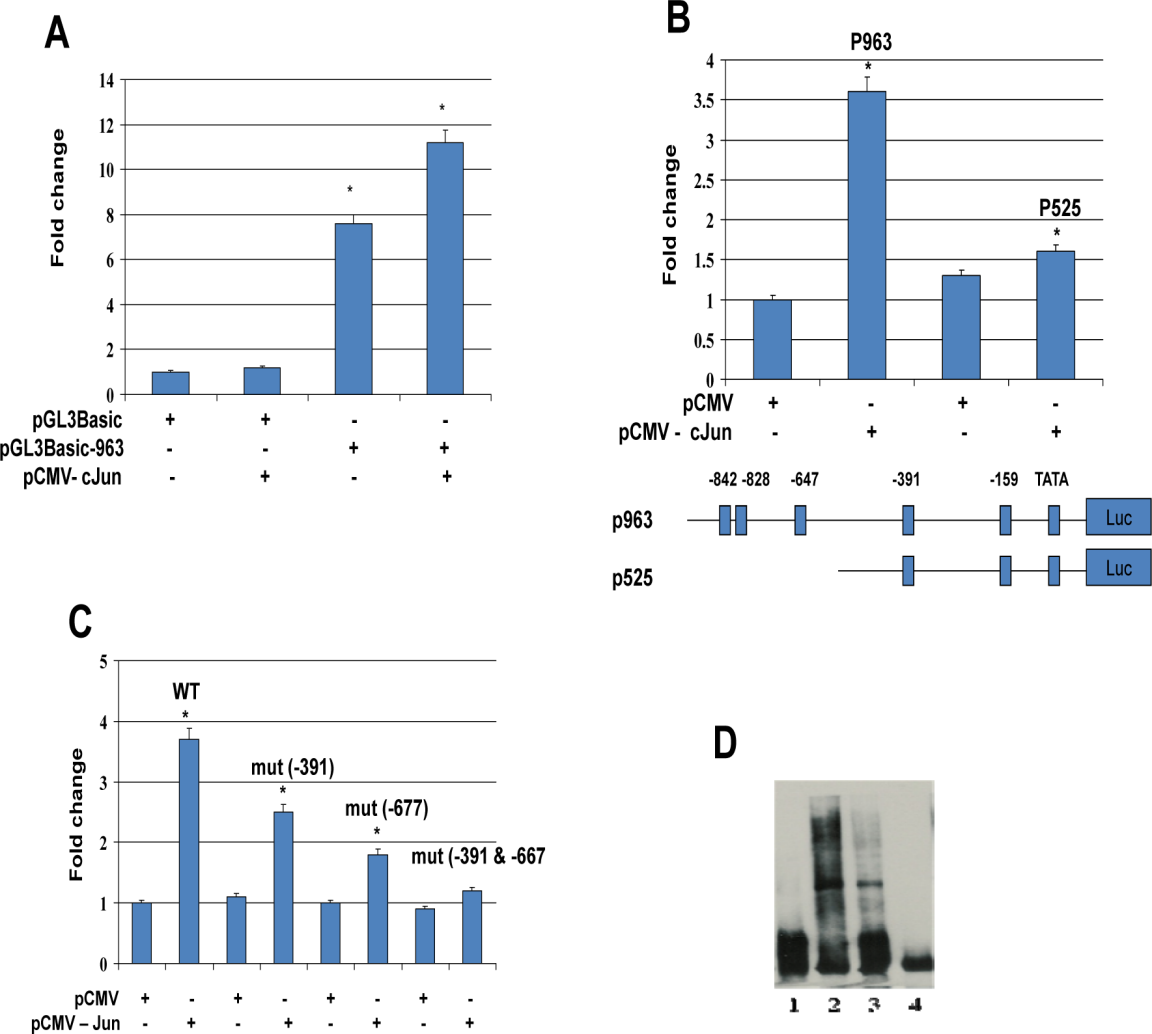
***TauT* is directly regulated by c-Jun.** Reporter genes were transiently transfected in MDCK cells co-transfected with or without c-Jun for 24 h, and then the luciferase assay was performed on cell lysates. A. Reporter gene pGL3-963, which contains two AP1 sites; B. Reporter gene pGL3-525, which contains one AP1 site; C. Reporter gene with double mutation of AP1 sites; D. Electrophoretic mobility shift assays were done using radiolabelled *TauT* oligonucleotides with nuclear extracts from Rat I cells expressing c-Jun. Lane 1 - control; lane 2 - wt *TauT* promoter DNA; lane 3 - single AP1 site deletion; and lane 4 - double AP1 site deletion. *p < 0.01 vs control.

Our studies have shown that *TauT* is regulated by several transcription factors, including Sp1, WT1, and p53 [14-16]. Figure 1 depicts the promoter region of *TauT* and the locations of potential binding sites for indicated transcription factors. Accordingly, several consensus sites for c-Jun/Ap1 are also found in the *TauT* promoter region. To determine the role of c-Jun in *TauT* expression, reporter genes were constructed and transiently transfected into MDCK kidney cells. As shown in Figure 2A, co-transfection of c-Jun enhanced *TauT* promoter activity (pGL3-963). Deletion of the site at -647 reduced the effect of c-Jun, and the effect was totally abolished when both -647 and -391 sites were mutated (Figure 2B &2C). Electrophoretic mobility shift assays showed that a strong c-Jun binding band was detected when both AP1 sites were presented in the probe. Deletion of the -647 site reduced abundance of the band (lane 3). Double deletion of AP1 sites abolished binding of c-Jun to the *TauT* promoter (lane 4, Figure 2D).

### Osmoregulation of *TauT* is negatively regulated by JNK

**Figure 3:**
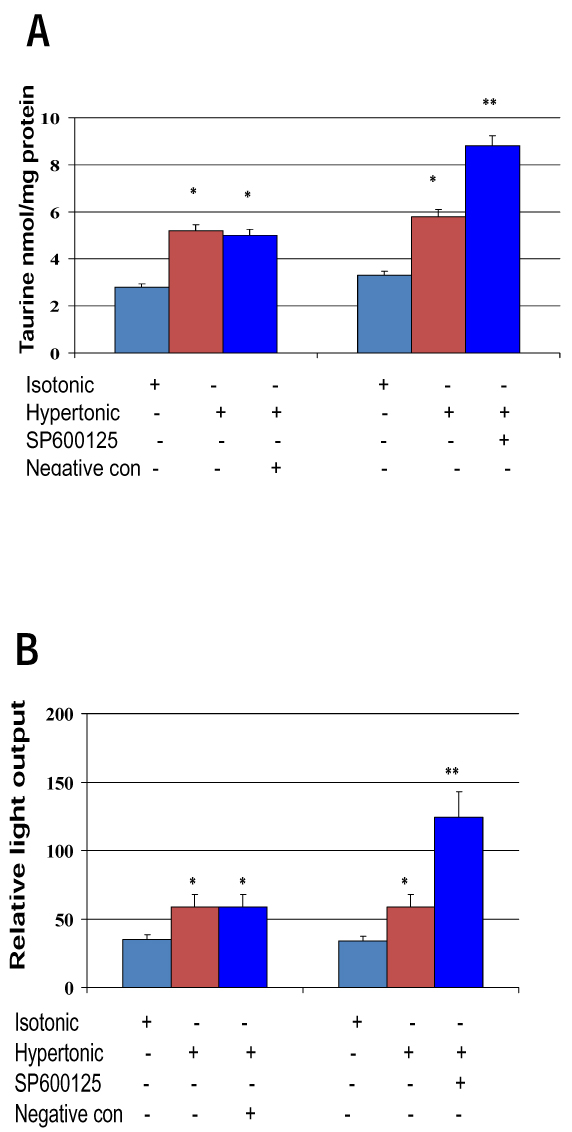
**Osmoregulation of *TauT* is mediated by JNK signaling pathway.** MDCK cells were cultured in DMEM medium and treated with 0 or 500 mosmol/kg JNK inhibitor SP600125 or negative control 420123 for 24 h. A. Taurine uptake; B Reporter gene assay. *p < 0.05 vs isotonic control; **p < 0.01 vs isotonic control.

To determine if JNK signaling is involved in osmoregulation of *TauT*, MDCK kidney cells were cultured in hypertonic medium (500 mosmol/kg) in the presence of JNK inhibitor SP600125 or negative control 420123 for 24 hours. MDCK cells cultured in normal medium were used as control. Expression of *TauT* was measured by taurine uptake and reporter gene expression. We found that taurine uptake was significantly increased in cells cultured in hypertonic medium. Inhibition of JNK by SP600125 further enhanced taurine uptake (Figure 3A). A similar result was observed in the reporter gene analysis in these cells (Figure 3B), suggesting that *TauT* was negatively regulated by JNK during hypertonic stress.

### *TauT* protects against cisplatin-induced AKI by blocking a p53-dependent pathway

**Figure 4:**
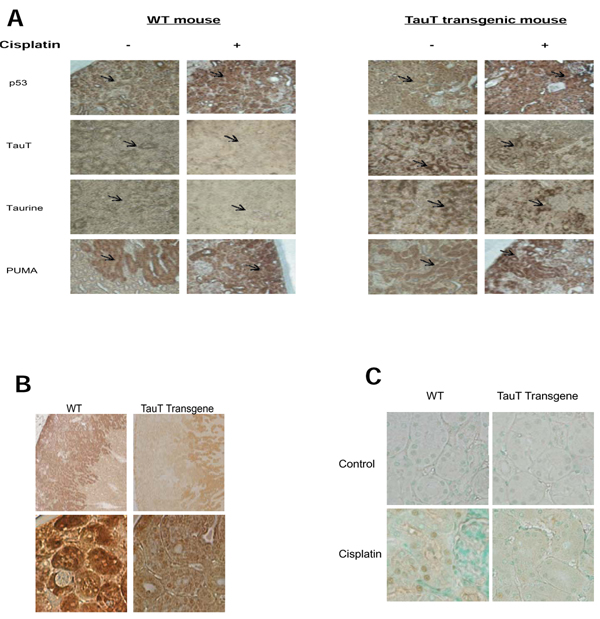
**Immunohistochemistry study of kidney tissues from wild-type and *TauT* transgenic mice with or without cisplatin treatment.** A. Immunostaining for p53, TauT protein, taurine, PUMA, and FasL (B) was performed on kidney tissue slides using specific antibodies against the indicated proteins as described in the Methods. C. TUNEL assay was performed on kidney tissue slides using a TACS TdT DAB *in situ* apoptosis detection kit (R&D system). Results represent three separate experiments (n=6). Magnification: A x 200, B (top) x 40 and (bottom) x 400, and C x 400.

Kidney tissues were examined by immunohistochemistry. As shown in Figure 4A, kidney tissue from *TauT* transgenic mice showed stronger immunostaining for *TauT* protein (TauT) and taurine as compared to that from wild-type (wt) mice. TauT and taurine were mainly found in the S3 segment of proximal tubule cells in normal wt animals, while TauT and taurine were also detected in the outer cortex stripe of *TauT* transgenic mice.

In saline-treated mice, p53 was detected in both proximal and distal tubular cells. Treatment with cisplatin increased expression of p53 to a similar degree in kidneys of both wt and transgenic animals. Cisplatin-induced activation of p53 down-regulated expression of *TauT* in the kidney from both wt and *TauT* transgenic mice, which in turn greatly reduced levels of intracellular taurine in the kidneys of both wt and *TauT* transgenic mice. However, both *TauT* and taurine were undetectable in cisplatin-treated wt mouse kidney, while signals of immunostaining for *TauT* and taurine in cisplatin-treated transgenic mouse kidney were still relatively high. In addition, expression of PUMA (p53-upregulated modulator of apoptosis) was also found in both proximal and tubular cells, and was elevated in both groups after cisplatin treatment. Interestingly, strong signals of immunostaining for PUMA were observed in the proximal tubules of the ourter cortex stripe, where vast apoptosis was observed in cisplatin-treated wt mice but not in cisplatin-treated *TauT* transgenic mice. We have also found that signals of immunostaining for FasL were higher in cisplatin-treated wt mice, which mainly co-located with p53 and PUMA in the outer stripe of kidney cortex, while FasL was only detected in the kidney medulla of *TauT* transgenic mice (Figure 4B). Moreover, TUNEL assay revealed clear evidence of cell death in proximal tubules of the cortex outer stripe (Figure 4C) of kidneys from cisplatin-treated wt mice, whereas few numbers of TUNEL-positive tubular epithelial cells were detected in the kidneys of cisplatin-treated *TauT* transgenic mice. Taken together, these results strongly suggest that *TauT* plays a critical role in protecting against cisplatin-induced AKI, possibly through blocking the p53-dependent pathway.

## Discussion

Studies have shown that *TauT* is regulated by variety of stresses, including tonicity, oxidation, DNA damage, and dietary manipulation [17-20], suggesting that *TauT* is a stress response gene. However, there are little data available about signaling pathway(s) that may be involved in stress-induced *TauT* regulation. In the present study we have demonstrated that *TauT* is a target of c-Jun. c-Jun binds to two AP1 consensus sites in the *TauT* promoter and up-regulates *TauT* expression in renal cells. Mutation of AP1 sites blocked binding of c-Jun to the *TauT* promoter and further abolished the effect of c-Jun on *TauT* regulation. Interestingly, we have found that expression of *TauT* as determined by taurine uptake is increased by SP600125, an inhibitor of JNK. It is possible that, binding of taurine to the transporter may contribute to the uptake result, which in turn may also indirectly indicate an increase of TauT on the cell membrane.  Taken together, our results suggest that the JNK signaling pathway is involved in the osmoregulation of *TauT* during hypertonic stress.

The JNK pathway is a major stress signaling pathway in cells that play important roles in many cellular processes, including development, apoptosis and cell growth. In non-stressed cells, JNK targets the ubiquitination and subsequent degradation of bound proteins such as c-Jun [21]. In addition, JNK forms a complex with and degrades p53 [22]. However, in stressed cells, JNK phosphorylates and activates associated c-Jun and p53 proteins and enhances their transcriptional regulation of stress-responsive genes [23,24]. Our previous studies have shown that *TauT* is a target gene of p53. Activation of p53 by a chemotherapeutic agent (cisplatin) suppresses *TauT* expression both *in vitro* and *in vivo *[18,25]. In the present study, we have further demonstrated that treatment with cisplatin increased expression of p53 to a similar degree in kidneys of both wt and transgenic mice. Cisplatin-induced p53 activation repressed *TauT* to an undetectable level, which in turn resulted in the depletion of taurine in the kidneys of wild-type mice. However, the levels of *TauT* and taurine in the kidneys of cisplatin-treated *TauT* transgenic mice are similar to those in the kidneys of normal control animals. Activation of p53 induced PUMA expression in both wt and *TauT* transgenic mice, although elevated levels of FasL were only observed in the kidneys of wt mice after cisplatin treatment. These findings suggest that relatively normal levels of *TauT* and/or taurine are able to protect against cisplatin-induced AKI.

**Figure 5:**
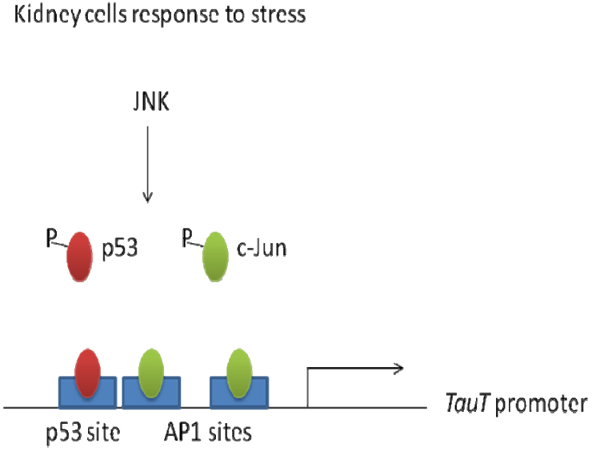
Stress-induced regulation of *TauT* by p53 and c-Jun via JNK signaling pathway

Based on these results, we propose a model for p53/c-Jun-mediated regulation of the *TauT* gene (Figure 5). Normally, both p53 and c-Jun bind to the *TauT* promoter and compete to regulate *TauT*. Therefore, the level of *TauT* protein is tightly regulated by p53 and c-Jun via the JNK signaling pathway. In stressed cells, JNK phosphorylates and activates c-Jun and p53 proteins and enhances their transcriptional regulation of *TauT*. Cisplatin-induced activation of p53 decreases *TauT* promoter activity via the p53-inhibited JNK-c-Jun pathway by competing with c-Jun for activation. Upon survival signaling, c-Jun substitutes for p53 function and enhances *TauT* expression. However, mechanisms of *TauT* regulation by p53/JNK-c-Jun pathway during stress-induced kidney injury remain to be determined.

## Conclusions

In the present study, we have shown that expression of *TauT* is negatively regulated by p53 and positively regulated by c-Jun, which is likely mediated by the JNK signaling pathway. Both p53 and c-Jun bind to and compete to regulate *TauT*. The ultimate level of *TauT* expression may determine the fate of renal cells during stress-induced AKI.

## List of abbreviations used

*TauT*: taurine transporter gene; AKI: acute kidney injury; JNK: c-Jun N-terminal kinase; RPTC: renal proximal tubular cell; AATC: American Association Tissue Culture; EBSS: Earle’s Balanced Salt Solution; TUNEL: terminal deoxynucleotidyl transferase (TdT)-mediated digoxigenin-deoxyuridine nick-end labeling.

## Competing interests

The authors declare that they have no competing interests.

## Authors' contributions

Authors contributed equally to this work
